# Comment on “Survival Improvement With Steroid Use for Pulmonary Veno‐Occlusive Disease With the Aid of Pulmonary Vasodilators and Tyrosine‐Kinase Inhibitor, a Retrospective Study”

**DOI:** 10.1002/clc.70430

**Published:** 2026-07-30

**Authors:** Wajeeha Khan, Muhammad Faizan Khan, Rafsha Khan

**Affiliations:** ^1^ Nowshera Medical College Nowshera Khyber Pakhtunkhwa Pakistan; ^2^ Khyber Medical University Peshawar Pakistan

To the Editor,

We read with great interest the article by Finger et al. [1], which concluded that prednisolone use was associated with improved survival in patients with pulmonary veno‐occlusive disease (PVOD). This work is particularly relevant given the absence of proven medical treatments for PVOD and its poor clinical prognosis. While we commend the authors for investigating a potential therapy in this challenging disease, several methodological limitations significantly weaken the strength of their conclusions and should be carefully considered when interpreting the results.

Our primary concern relates to the statistical fragility of the study's principal finding. It rests on a log‐rank *p* = 0.01 derived from only five patients per group, making this result highly fragile. Notably, three patients in the steroid group remained alive at the study cutoff; consequently, the reported statistical significance depends on the outcomes of only a few individuals and may therefore be highly unstable. If even one of these patients had died earlier, the difference may no longer have reached statistical significance [2]. Notably, the authors' own data acknowledge this fragility implicitly, as three of the five steroid‐treated patients had not reached the outcome event at the time of analysis, leaving the result dependent on follow‐up duration as much as treatment effect. Evidence based on such a limited number of events provides a weak basis for inference, as small changes can substantially alter the results. In this context, the *p* value is more likely to reflect the constraints of a very small sample than true efficacy.

Two additional concerns further weaken the study's conclusions. First, epoprostenol exposure was starkly unequal between groups, used in four of five non‐steroid patients but none of the steroid‐treated patients, an imbalance the original authors themselves acknowledge as a possible contributor to the non‐steroid group's worse prognosis [3]. Epoprostenol is not a neutral drug and carries a well‐recognized risk of pulmonary edema in PVOD patients. Consequently, differential epoprostenol‐related harm in the non‐steroid group may have independently worsened their outcomes, creating a false appearance of prednisolone efficacy rather than reflecting any true therapeutic benefit.

Second, the groups were treated during different time periods (2002–2006 vs. 2006–2018), separated by more than a decade of clinical progress. Over this period, advances in clinical practice and supportive care are likely to have influenced patient outcomes [4]. This period coincided with the introduction of EIF2AK4 mutation screening and expanded transplant referral pathways, both of which may independently improve survival in PVOD patients. While the authors argue that no major treatment advance occurred during this interval and that background characteristics were otherwise similar between groups, this defense does not address whether diagnostic, referral, or supportive care practices changed over a 16‐year span, nor does it account for genetic stratification, since EIF2AK4 mutation status was not assessed in either group.

In conclusion, the statistical fragility, pharmacological confounding, and temporal heterogeneity outlined above preclude any causal inference regarding prednisolone efficacy in this cohort. Future studies should prioritize prospective, registry‐based designs with propensity‐matched controls, standardized concomitant therapy protocols, and predefined survival endpoints. Given the rarity of PVOD, international multicenter collaboration and stratification by EIF2AK4 mutation status would be essential to ensure adequate power and biological homogeneity.

## Author Contributions

All authors have read and approved the final manuscript.

## Funding

The authors have nothing to report.

## Ethics Statement

The authors have nothing to report.

## Consent

The authors have nothing to report.

## Conflicts of Interest

The authors declare no conflicts of interest.

## Data Availability

The authors have nothing to report.
